# Modern Approaches to the Synthesis and Transformations of Practically Valuable Benzothiazole Derivatives

**DOI:** 10.3390/molecules26082190

**Published:** 2021-04-10

**Authors:** Larisa V. Zhilitskaya, Bagrat A. Shainyan, Nina O. Yarosh

**Affiliations:** E. Favorsky Irkutsk Institute of Chemistry, Siberian Branch of the Russian Academy of Sciences, 1 Favorsky Street, 664033 Irkutsk, Russia; lara_zhilitskaya@irioch.irk.ru (L.V.Z.); yarosh.nina@irioch.irk.ru (N.O.Y.)

**Keywords:** benzothiazole, 2-aminobenzothiazole, 2-mercaptobenzothiazole, synthesis, reactivity, biological activity

## Abstract

The review is devoted to modern trends in the chemistry of 2-amino and 2-mercapto substituted benzothiazoles covering the literature since 2015. The reviewed heterocycles belong to biologically active and industrially demanded compounds. Newly developed synthesis methods can be divided into conventional multistep processes and one-pot, atom economy procedures, realized using green chemistry principles and simple reagents. The easy functionalization of the 2-NH_2_ and 2-SH groups and the benzene ring of the benzothiazole moiety allows considering them as highly reactive building blocks for organic and organoelement synthesis, including the synthesis of pharmacologically active heterocycles. The review provides a summary of findings, which may be useful for developing new drugs and materials and new synthetic approaches and patterns of reactivity.

## 1. Introduction

Benzothiazoles, as a bicyclic heterocycles with fused benzene and thiazole rings containing electron-rich heteroatoms, nitrogen and sulfur, attract great interest from researchers for drug design due to their high biological and pharmacological activity [1,2,3,4,5]. The present review covers the literature from 2015. We consider modern trends in synthesizing biologically active and industrially demanded compounds based on the C-2-substituted benzothiazole derivatives. The reactions of 2-amino- and 2-mercaptothiazole derivatives provide a powerful, modern tools for the design of a wide variety of aromatic azoles. Their synthesis methods can be divided into two main groups: “one-pot” synthesis and sequential multistep synthesis.

Along with conventional approaches, effective and ecologically friendly alternative reactions are being developed based on commercially available reagents and the principles of green chemistry. These avoid the use of toxic solvents and minimize the formation of side products. Many reactions are performed in water as the solvent, making the process much cheaper. Multistep one-pot reactions of the C-2-substituted benzothiazoles play a special role in the design of biologically active compounds. The advantages of these reactions are atom-economy, simple experimental implementation and high yields. The effectiveness and selectivity of multistep reactions can often be increased by using catalysts in the absence of solvents. In addition, the methods based on the combined use of microwave irradiation and multicomponent reaction in ecologically safe solvents or in the solvent-free mode are actively used nowadays.

## 2. Aminobenzothiazoles

2-Aminobenzothiazoles demonstrate high antiviral activity [6,7,8,9,10,11,12,13,14], which is especially important in this period of global COVID-19 pandemic. They also possess antimicrobial [14,15,16,17,18,19,20], antioxidant [20], anti-inflammatory [21,22,23,24], analgesic [24], antidepressant [25,26], anticonvulsant [27,28,29], anti-diabetic [21,30], antitumor [31,32,33,34,35], antituberculosis [14,36] activity. Their derivatives show also antimalarial [37,38], insecticide [39] and herbicide effect [40]. They are also used as key intermediates in fine organic synthesis and the resins’ components [41].

### 2.1. Synthesis

Several approaches to the thiazole ring formation are based on transition metal catalysis using a one-pot process, either solvent-free or in green solvents.

**Scheme 1:**
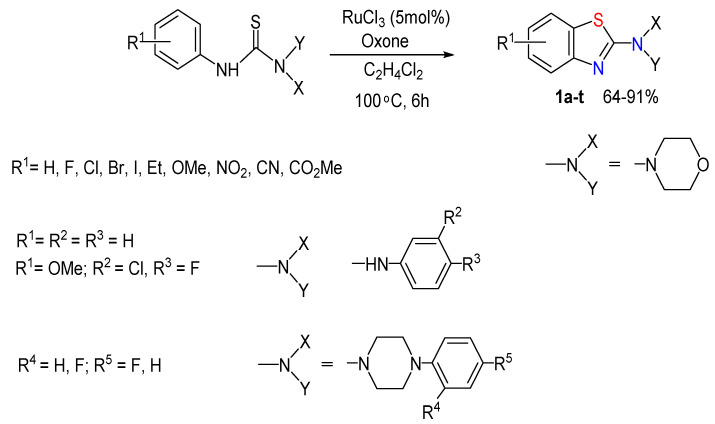
Ru(III)-catalyzed synthesis of substituted 2-aminobenzothiazoles from *N*-arylthioureas.

Thus, direct synthesis of the substituted 2-aminobenzothiazoles **1a**–**t** via the RuCl_3_-catalyzed intramolecular oxidative coupling of *N*-arylthioureas in up to 91% yield was proposed (Scheme 1) [42]. The electron-rich substrates demonstrate higher reactivity than their electron-deficient analogs.

**Scheme 2:**
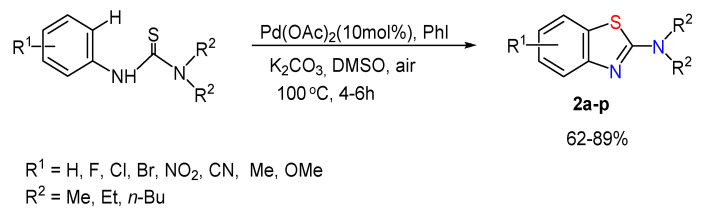
Pd-catalyzed synthesis of 2-(dialkylamino)benzothiazoles from *N*-aryl-*N’*,*N’*-dialkylthioureas.

The Pd(OAc)_2_-catalyzed intramolecular oxidative cyclization of *N*-aryl-*N′*,*N′*-dialkylthioureas proceeds similarly, resulting in the formation of 2-(dialkylamino)benzothiazoles **2a**–**p** in the same yields (Scheme 2) [43]. No products of the intermolecular coupling were formed. However, in view of preactivation, more expensive catalyst and its larger amount, the method is more costly.

**Scheme 3:**
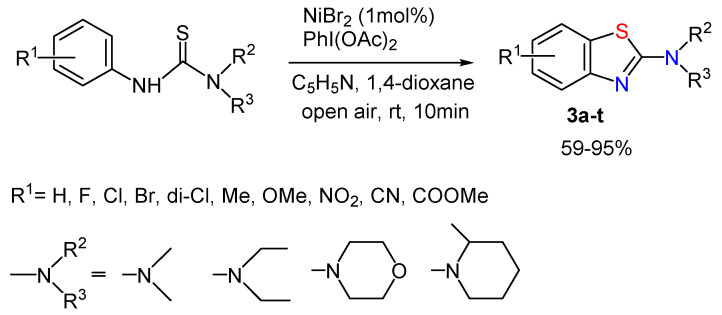
Ni(II)-catalyzed synthesis of 2-aminobenzothiazoles **3a**–**t** from *N*-arylthioureas.

Very recently, Ni(II) salts were shown to be very good catalysts for the same reaction [44]. The method is more advantageous as it uses a cheaper and less toxic catalyst in a much lower concentration, and the reaction is carried out under mild conditions in a very short time resulting in up to 95% product yield (Scheme 3). The method can be applied to *N*-arylthioureas containing both electron-donating or electron-withdrawing substituents in the benzene ring and is scalable without any loss of the yield, which makes it promising for industrial application.

**Scheme 4:**
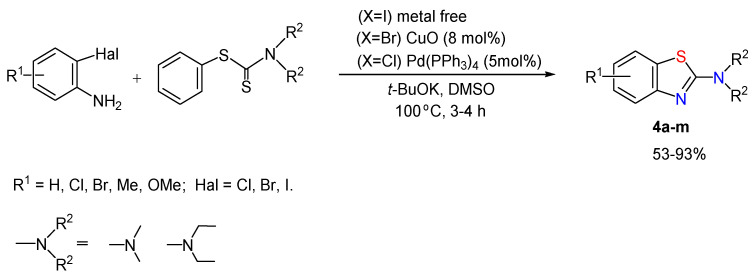
Synthesis of 2-aminobenzothiazoles **4a**–**m** from 2-haloanilines and dithiocarbamates.

An alternative intermolecular approach to 2-aminobenzothiazoles **4a**–**m** is based on the reaction of 2-haloanilines with dithiocarbamates (Scheme 4) [45]. The metal-free or transition-metal-catalyzed reaction proceeds in one pot in up to 93% yield of the products. Thus, the reaction of 2-iodoaniline with dithiocarbamate proceeds without a catalyst, whereas 2-bromoanilines react only in the presence of copper catalysts, among which CuO was found to be most effective. Due to the low reactivity of the C_Ar_–Cl bond, 2-chloroanilines were even less reactive and required more strong catalyst Pd(PPh_3_)_4_. Note that such palladium catalysts as PdCl_2_, PdBr_2_ or Pd(OAc)_2_ showed poor catalytic activity, and the copper catalysts were ineffective. The reaction proceeds in two steps. In the first step, the base (*t*-BuOK) promotes the formation of arylthiourea, which is converted to the arylisothiourea. In the second step, either metal-free or transition-metal-catalyzed intramolecular cross-coupling occurs with the formation of the target 2-aminobenzothiazoles.

**Scheme 5:**
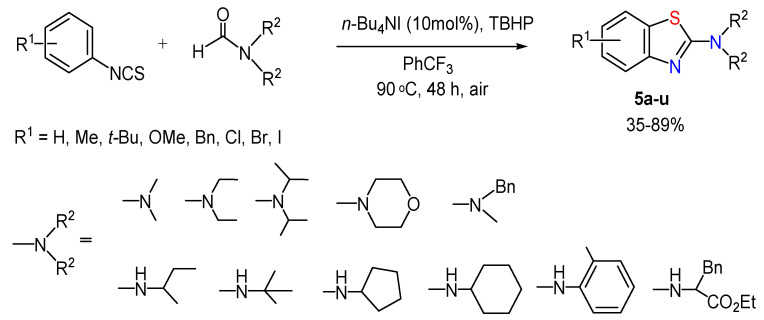
Synthesis of 2-aminobenzothiazoles **5a**–**u** from arylisothiocyanates and formamides.

The metal-free reactions of sulfanylation of the C–H bond are most appealing and in line with the principles of green chemistry. Isothiocyanates are universal building blocks used to synthesize various heterocyclic ensembles, mostly triggered by nucleophilic addition. A convenient synthesis of 2-aminobenzothiazoles **5a**–**u** from readily available arylisothiocyanates and various formamides was reported (Scheme 5) [46]. The reaction was assumed to proceed as decarbonylation of formamide under the action of *n*-Bu_4_NI and *t*-BuOOH (TBHP) and formation of the aminyl radical, its addition to the isothiocyanate and subsequent cyclization of the S-centered radical intermediates. The yield is strongly affected by the nature and the position of the substituent in the phenyl ring.

**Scheme 6:**
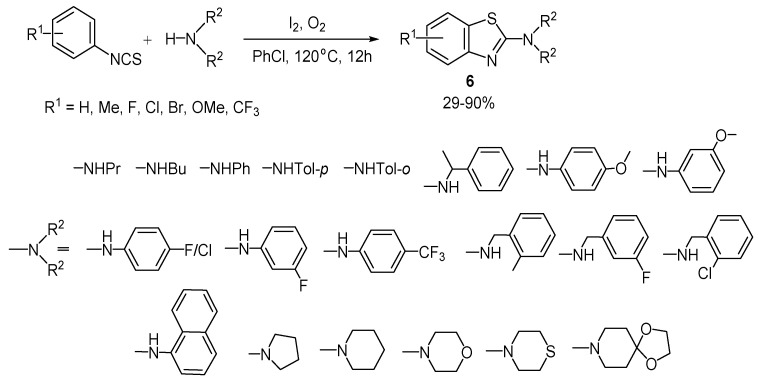
Synthesis of 2-aminobenzothiazoles from arylthioisocyanates and amines.

Arylisothiocyanates also enter the one-pot cascade reaction with amines in the presence of iodine as the catalyst and molecular oxygen as the oxidant (Scheme 6) [47]. The reaction proceeds via the in situ formation of benzothiourea with its subsequent intramolecular oxidative cyclization. Inexpensive and ecologically pure oxidant, commercially available reagents, non-toxic side product, and no necessity to introduce a halogen to the ortho-position—all these advantages meet the criteria of green chemistry. The yield of the products is higher for the compounds with the electron-donating groups in the benzene ring.

**Scheme 7:**
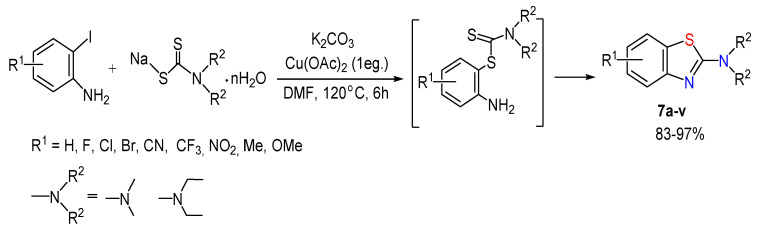
Synthesis of 2-aminobenzothiazoles **7a**–**v** from iodoanilines and sodium dithiocarbamates.

A simple and effective one-pot synthesis of 2-aminobenzothiazoles 7 from 2-iodoanilines and sodium dithiocarbamates by the Ullmann-type reaction (C_Ar_–S bond formation via the intermediate copper thiolate) was developed with the yields of the products reaching 97% (Scheme 7) [48]. Cu(OAc)_2_/Cs_2_CO_3_/DMF/120 °C was found to be the best combination of the copper(II) catalyst, base, solvent, and temperature. The electron-donating or withdrawing properties of the substituents in the ring do not affect the products’ yield, reaching 97% for 2-iodo-4-fluoroaniline. The method employs readily available reagents, inexpensive ligand-free catalyst, provide good yields of potentially biologically active compounds and, therefore, is practically valuable.

**Scheme 8:**
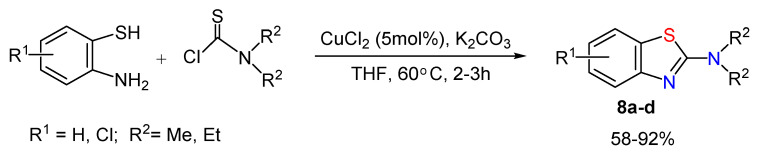
Synthesis of 2-aminobenzothiazoles **8a**–**d** from aminothiophenols and thiocarbamoyl chloride.

The formation of 2-aminobenzothiazoles **8a**–**d** is possible also by one-pot condensation of aminothiophenols with thiocarbamoyl chloride in the presence of CuCl_2_ and K_2_CO_3_ proceeding under mild conditions in good yields (Scheme 8) [49].

**Scheme 9:**
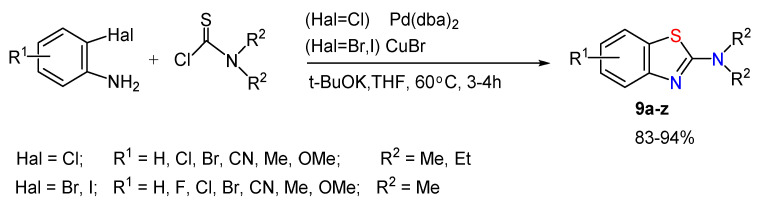
Synthesis of 2-aminobenzothiazoles **9a**–**z** from haloanilines and thiocarbamoyl chloride.

Thiocarbamoyl chloride also reacts effectively with 2-bromo or 2-iodoanilines in the presence of CuBr and *t*-BuOK to afford the corresponding 2-aminobenzothiazoles **9a**–**z** in good yields (Scheme 9) [50]. Less reactive 2-chloroanilines do not enter this reaction. The reaction starts with the base-promoted formation of arylthioureas, which were further converted to arylisothioureas. In the next step, the target products are formed by the reaction of intramolecular cross-coupling. The method is characterized by mild reaction conditions, inexpensive catalyst, good yield and a wide scope of substrates. 2-Chloroanilines and thiocarbamoyl chloride afford 2-aminobenzothiazoles only in the presence of such a strong catalyst as bis(dibenzylideneacetone)palladium(0) Pd(dba)_2_ (Scheme 9) [51].

**Scheme 10:**
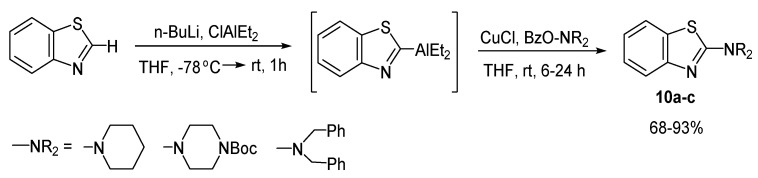
Synthesis of 2-aminobenzothiazoles **10a**–**c** from benzothiazole and *O*-benzoylhydroxylamines.

An alternative route to 2-aminobenzthiazoles is direct aluminum organic synthesis shown in Scheme 10 [52]. The intermediate organoaluminum compound formed by lithiation/alumination of benzothiazole reacts with *O*-benzoylhydroxylamines. The copper-catalyzed reaction proceeds in one pot under mild conditions in up to 93%.

**Scheme 11:**
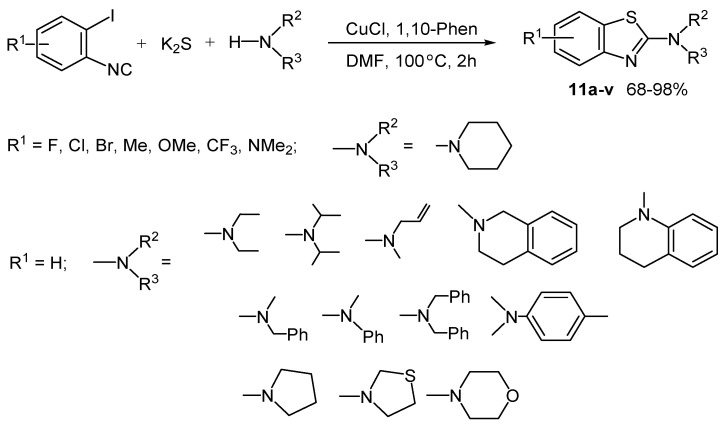
Three-component synthesis of 2-aminobenzothiazoles **11a**–**v**.

The three-component copper-catalyzed reaction of 2-iodophenylisocyanides, potassium sulfide and various amines was shown to give biologically interesting 2-aminobenzothiazoles in up to quantitative yield (Scheme 11, the optimal conditions obtained by screening of the alkali metal, catalyst, solvent, and temperature are given) [53].

### 2.2. Alkylation and Acylation Reactions

Due to the presence of both exo and endo nitrogen atoms forming the amidine–N=C–NH_2_ motif, 2-aminobenzothiazoles can enter the reactions of alkylation and acylation of either the amino group or annulation with the whole amidine fragment being involved in giving various heterocycles with fused rings. Below, we will consider some of these reactions.

**Scheme 12:**
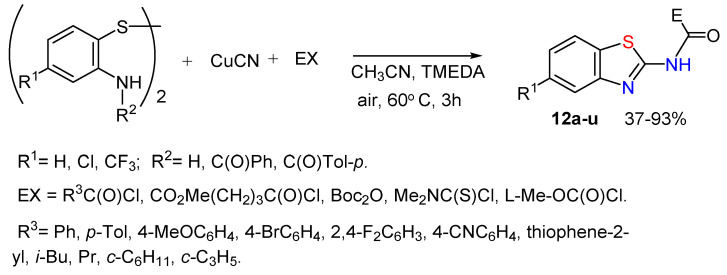
Synthesis of the ring-substituted *N*-acylated 2-aminobenzothiazoles **12a**–**u**.

The ring-substituted 2-aminobenzothiazoles formed in situ from diaminodiaryl disulfides enter the three-component domino reaction, which includes diacylation of diaminodiaryl disulfide, oxidative S-cyanation by CuCN, cyclization via nucleophilic attack of the thiocyanate carbon atom and, finally, intermolecular acyl group migration to the exocyclic nitrogen atom (Scheme 12) [54].

**Scheme 13:**
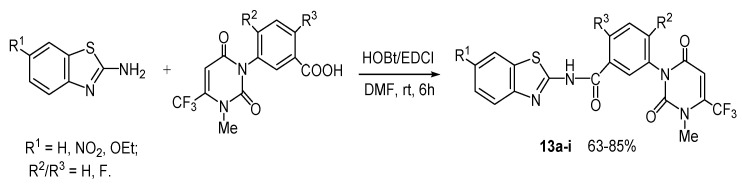
Synthesis of the uracil-containing *N*-benzothiazolyl benzamides **13a**–**i** with herbicide activity.

Acylation of 6-substituted 2-aminobenzothiazoles by polysubstituted benzoic acids containing the uracil moiety affords benzamides **13a**–**i** possessing herbicide activity (Scheme 13) [40].

**Scheme 14:**
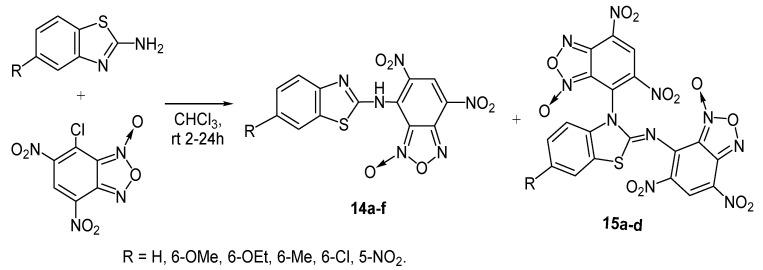
Mono- and dialkylation of 2-aminobenzthiazoles by 7-chloro-4,6-dinitrobenzofuroxane.

Alkylation of 2-aminobenzothiazoles is possible by highly reactive halogenides, such as, e.g., 7-chloro-4,6-dinitrobenzofuroxane having two strong activating nitro groups [19]. First, the primary amino group is alkylated to give the monoalkylated products **14a**–**f**, which after tautomerization give the dialkylated products **15a**–**d** (Scheme 14). Remarkably, for the substrates containing electron-withdrawing substituents (6-Cl, 6-NO_2_), the dialkylation products were not formed. It was noted that in time the monoalkylated product became the major one. The obtained compounds were shown to have antimicrobial activity and also can be used as luminescing biosensors.

**Scheme 15:**
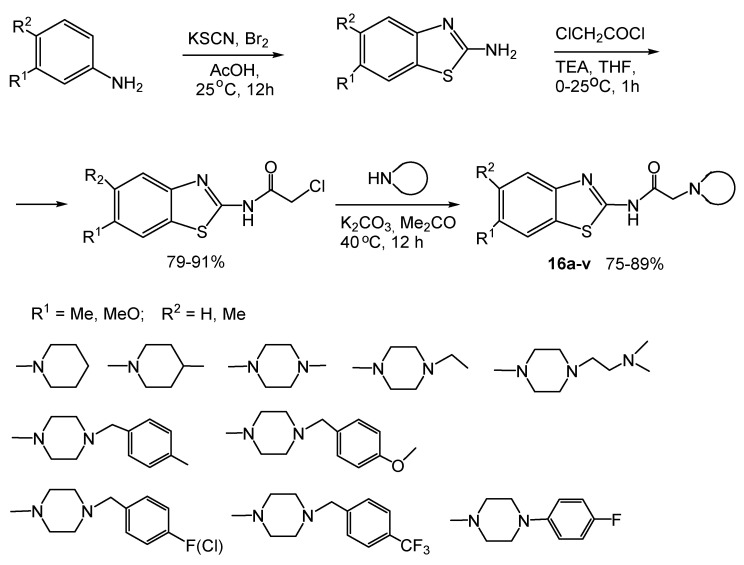
Synthesis of antidepressant heterocycles **16a**–**v**.

Many researchers were engaged in the search for new families of biologically active 2,5(6)-substituted 2-aminobenzothiazoles. The starting 2-unsubstituted 2-aminobenzothiazoles were obtained from the easily available anilines, potassium thiocyanate and bromine, then acylated by chloroacetyl chloride and introduced in the reactions with various *N*-heterocycles [7,14,17,20,25,26,27,28,29,31,36,38,39]. Thus, a large benzothiazole-piperazine series of compounds **16a**–**v** was synthesized in three steps (Scheme 15) [25,26]. The compounds demonstrated good antidepressant activity without accompanying negative effects on physical activity.

**Scheme 16:**
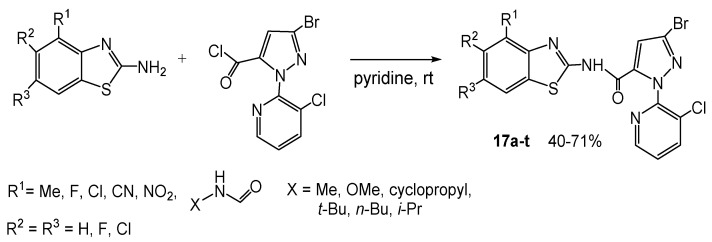
Synthesis of benzothiazolyl-piperazine heterocycles **17a**–**t** with insecticide activity.

Similarly prepared 4,5,6-trisubstituted 2-aminobenzothiazoles were acylated by 3-bromo-1-(3-chloropyridin-2-yl)-1*H*-pyrazole-5-carbonyl chloride to give *N*-1,3-benzothiazol-2-yl-3-bromo-1-(3-chloropyridin-2-yl)-1*H*-pyrazole-5-carboxamides **17a**–**t** (Scheme 16), which showed insecticide activity [39].

**Scheme 17:**
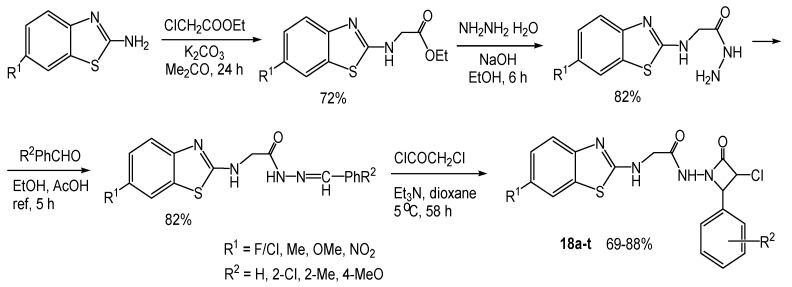
Synthesis of polyheterocyclic acetamides **18a**–**t**.

6-Substituted 2-aminobenzothiazoles prepared in the same manner were treated first with ethyl chloroacetate and then with hydrazine hydrate to afford the corresponding acetohydrazides. The latter’s condensation with aldehydes led to benzylideneacetohydrazides, which after acylation with chloroacetyl chloride gave acetamides **18a**–**t** (Scheme 17). The products were tested for the anticonvulsant effect, and those containing electron-withdrawing groups (Cl, F, NO_2_) in the benzothiazole ring and unsubstituted or chlorine-substituted in the benzene ring were found to be more active [28].

**Scheme 18:**
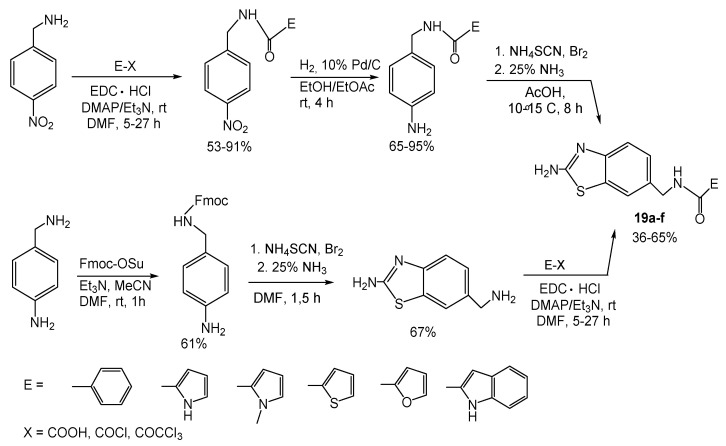
Multistep syntheses of 2-aminobenzothiazole analogs of clathrodine **19a**–**f**.

Multistep synthesis of 2-aminobenzothiazole analogs of clathrodine (marine pyrroloiminoquinone alkaloid) **19a**–**f** was reported (Scheme 18) [7]. *N*-Acylated 4-nitrobenzylamines were reduced to the corresponding anilines, which were cyclized by ammonium thiocyanate to the target products. However, the yields in the last step for the azole electrophiles were too low (0–10%) (Scheme 18). The alternative approach using *N*-protection of 4-aminobenzylamine by Fmoc as the first step (lower line in Scheme 18) allowed obtaining the products via the intermediate 6-aminomethyl-2-aminobenzothiazole in 36–54%. The antimicrobial, antiviral and antiproliferative activity of the products was estimated.

**Scheme 19:**
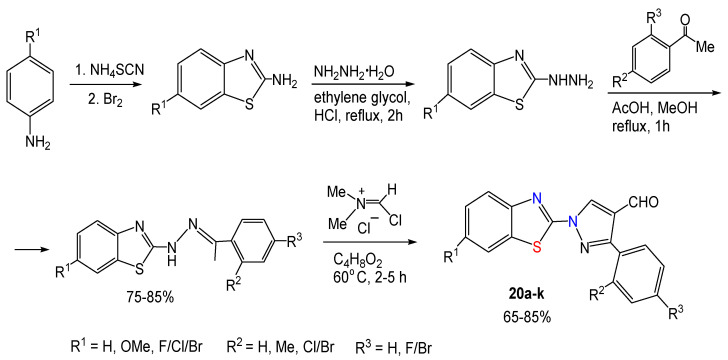
Synthesis of 1-benzothiazolyl-3-aryl-4-formyl-1*H*-pyrazoles **20a**–**k**.

6-Substituted 2-aminobenzotriazoles obtained by the same procedure (with NH_4_SCN and Br_2_) were successively subjected to hydrazinolysis and reacted with acetophenones to obtain the corresponding hydrazones. The latter was converted to the target products **20a**–**k** in 65–85% by the Vilsmeier–Haack reaction (Scheme 19) [20]. The reaction proceeds smoothly for the electron releasing or withdrawing substituents in the *o*- and *p*-positions of the phenyl ring and 6-position of the benzothiazole ring.

**Scheme 20:**
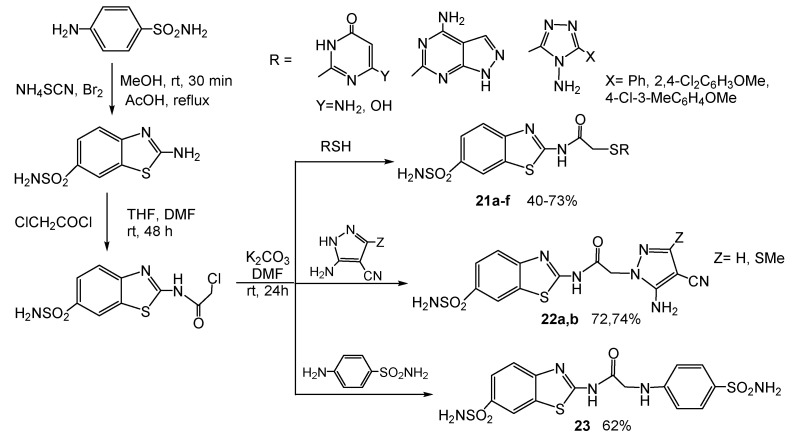
Synthesis of benzothiazole-containing sulfanilamide derivatives **21**–**23**.

The ammonium thiocyanate-based method was applied to synthesize a large series of 2-aminobenzothiazoles **21**–**23** containing the motif of sulfanilamide, which laid the foundation for a large family of sulfa antimicrobial drugs. The obtained aminobenzo[*d*]thiazole-6-sulfonamide was acylated with chloroacetyl chloride, which then reacted with thiols to give products **21**, or with 5-amino-1*H*-pyrazole-4-carbonitrile to give benzothiazolo-pyrazole heterocycles **22**, or with another molecule of sulfanilamide resulting in compound **23** with two pharmacophore sulfanilamide moieties (Scheme 20) [31]. All products showed high antitumor activity.

**Scheme 21:**
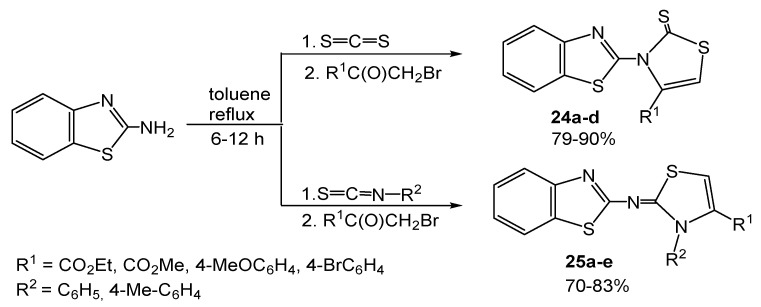
Synthesis of functionalized bis-thiazolo derivatives **24a**–**d** and **25a**–**e**.

Acylation of 2-aminobenzothiazole with various α-halogenoketones in the presence of carbon disulfide or arylisothiocyanates led to functionalized bis-thiazolo derivatives **24a**–**d** and **25a**–**e** in high yields (Scheme 21) [55]. Their formation proceeds via the addition of carbon disulfide to 2-aminobenzothiazole, acylation, enolization of the formed intermediate and cyclization with dehydration.

**Scheme 22:**
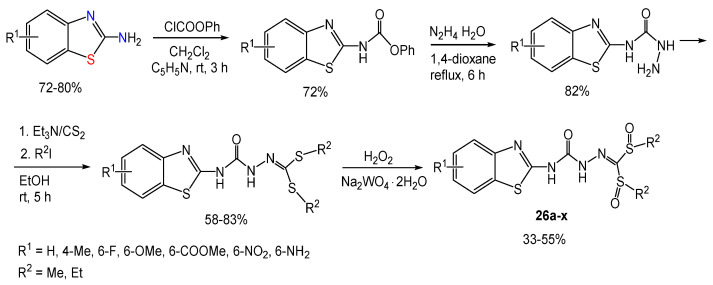
Multistep synthesis of benzothiazole/semicarbazone/disulfoxide-containing products **26a**–**x** with antibacterial properties.

Very recently, starting with acylation of the ring-substituted 2-aminobenzothiazoles, and followed by hydrazinolysis, thioetherification with carbon disulfide and oxidation, a large family of bis-sulfoxide derivatives **26a**–**x** containing the acylhydrazone and the benzothiazole groups have been synthesized (Scheme 22) [56] and shown to possess antibacterial activity.

**Scheme 23:**
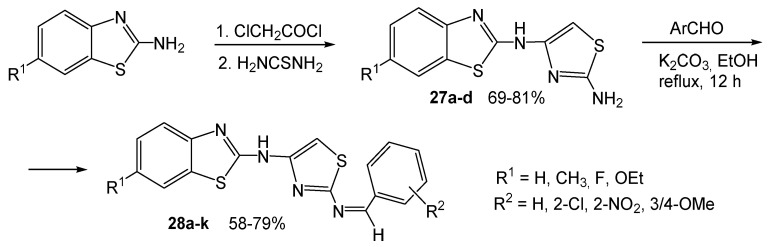
Synthesis of bis-thiazolo-containing amines **27a**–**d** and azomethines **28a**–**k**.

A similar approach was earlier used to synthesize heterocyclic amines **27a**–**d** and their Schiff bases **28a**–**k** [17]. Acylation with chloroacetyl chloride followed by cyclization with thiourea to form the second 2-aminothiazole moiety and condensation of the amino group in amines **27a**–**d** with aromatic aldehydes led to the target azomethines **28a**–**k** in moderate yields (Scheme 23). The products were tested on the biological activity, and both amines **27a**–**d** and the Schiff bases **28a**–**k** showed high or even maximum antibacterial, antifungal and anthelminthic activity. Electron-donor groups (Me, OEt) in the 6-position of the benzothiazole ring enhance the anthelminthic effect. The NO_2_ group in the phenyl ring promotes antibacterial activity. Electron-acceptor F and Cl atoms both in the benzothiazole and the phenyl ring enhance antifungal activity.

**Scheme 24:**
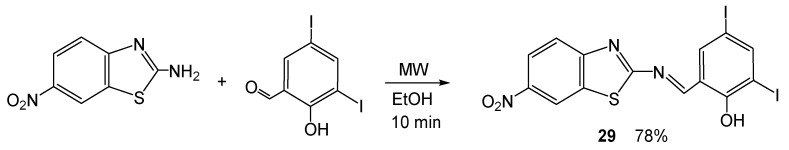
Schiff base **29** from 2-amino-6-nitrobenzothiazole and 3,5-diiodosalicylic aldehyde.

The Schiff base **29** formations between the NH_2_ group of 2-amino-6-nitrobenzothiazole was performed by the MW-assisted reaction with a 3,5-diiodosalicylic aldehyde in 76–80% yield in 8–10 min (Scheme 24) [57]. For example, without MW irradiation, the yield was as low as 38% after 2 h.

**Scheme 25:**
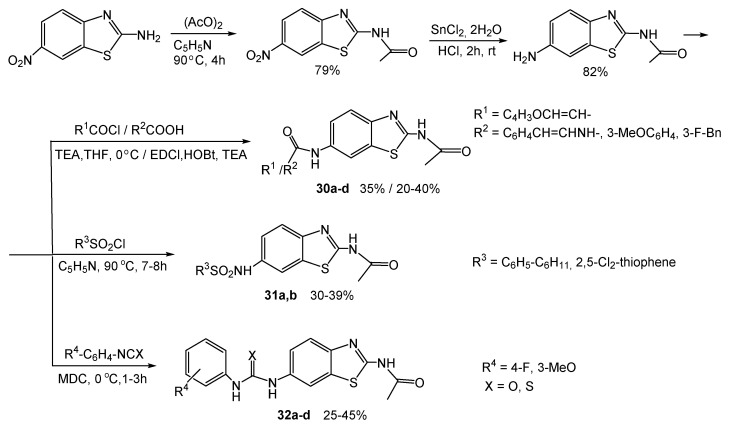
Synthesis of 6-*N*-functionalized derivatives of 2-aminobenzothiazoles **30**–**32**.

The synthesis of the derivatives of 2-aminobenzothiazoles with *N*-functionalized groups in the benzene ring is possible from the corresponding nitro-derivatives by pre-protection of the 2-amino group followed by reduction of the nitro to the amino group and its functionalization. Using this approach compounds **30**–**32** with high anti-inflammatory activity were synthesized in three steps from 6-nitro-2-aminobenzothiazole, as shown in Scheme 25 [21]. Different functions have been introduced by the reactions of acylation, sulfonylation or addition to isocyanates of isothiocyanates.

**Scheme 26:**
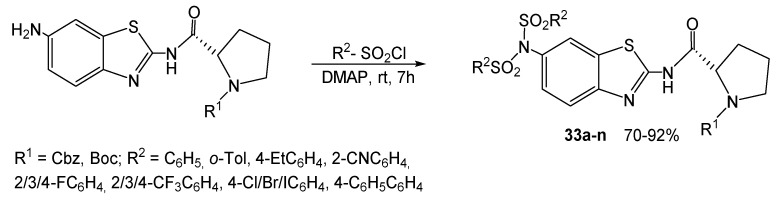
Synthesis of new benzothiazole-disulfonamide scaffolds **33a**–**n** as viral inhibitors.

A similar ideology was employed to synthesize new benzothiazol-disulfonamide scaffolds **33a**–**n** as a potent hepatitis C virus inhibitor (Scheme 26) [58]. The key intermediate product prepared by the reaction of 6-nitro-2-aminobenzothiazole with proline activated by *N-*(3-dimethylaminopropyl)-N′-ethylcarbodiimide (EDCl) with subsequent reduction of the nitro group (omitted in Scheme 26) is sulfonylated by arenesulfonyl chlorides at the free amino group. The obtained aminobenzothiazoles have a smaller molecular mass and hydrophobicity concerning the known antiviral agents, which provides better peroral bioavailability.

**Scheme 27:**
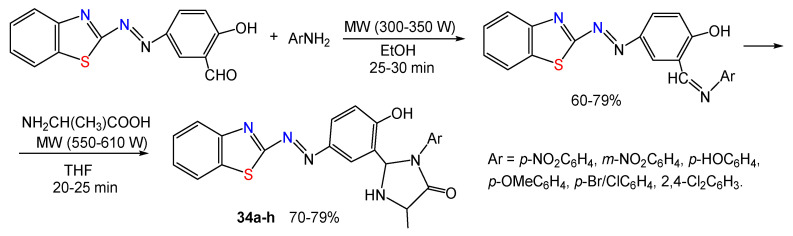
Synthesis of benzothiazole-imidazolidines **34a**–**h**.

MW irradiation was also used in the synthesis of benzothiazole-imidazolidines **34a**–**h**. The diazonium salt of 2-aminobenzothiazole reacted with a salicylic aldehyde in an alkaline solution to form the azoaldehyde derivative of benzothiazole [15]. The latter was cyclized by condensation with primary aromatic amines under MW-irradiation, and the formed imines were reacted with L-alanine under MW-irradiation in THF to give imidazolidines with benzothiazolyl fragment possessing antibacterial activity (Scheme 27).

**Scheme 28:**
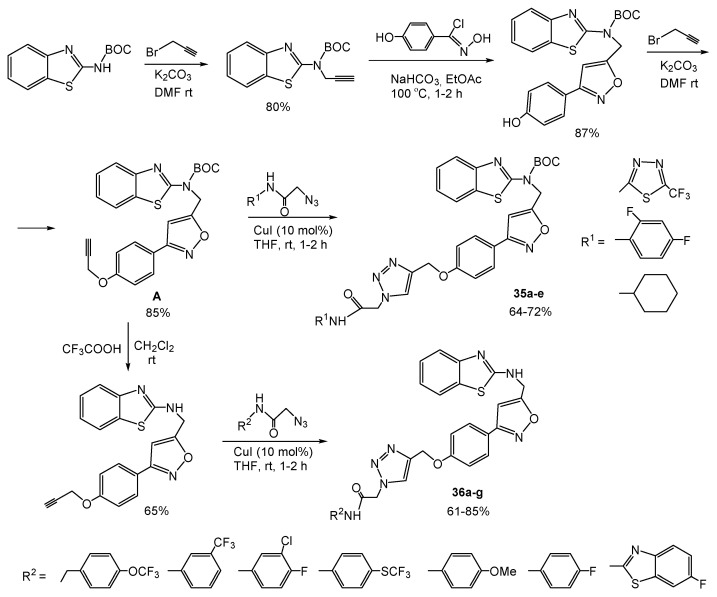
New potent cytotoxic hybrid azolyl derivatives of benzothiazole **35a**–**c** and **36a**–**g**.

Triazole and isoxazole-tagged benzothiazole/benzoxazole derivatives **35a**–**c** and **36a**–**g** were synthesized as potent cytotoxic agents by multistep synthesis starting from *N*-propargylation of the Boc-protected 2-aminobenzothiazole, as shown in Scheme 28 [33]. The key intermediate A was converted to the target products, either directly or after deprotection with trifluoroacetic acid, using Cu-catalyzed Sharpless click chemistry concept by the reaction with different amide azides. The presence of various pharmacophores in the synthesized compounds not only enhances the antitumor effect in vivo but also promotes new mechanisms of action.

### 2.3. Annulation Reactions

**Scheme 29:**
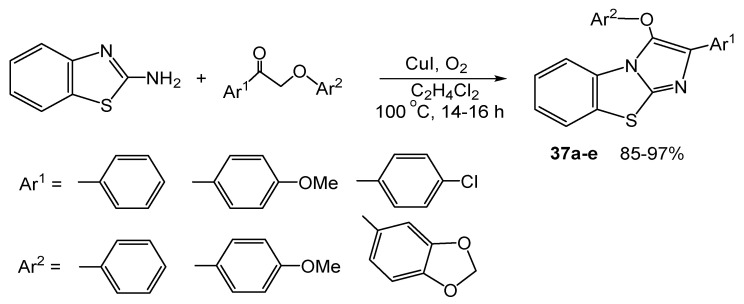
Synthesis of 3-phenoxybenzo[*d*]imidazo[2,1-*b*]thiazoles **37a**–**e**.

In a number of works, both nitrogen atoms of the amidine moiety in 2-aminobenzothiazole are involved in the reaction with electrophiles, resulting in the formation of complex heterocyclic systems with fused rings. Thus, CuI-catalyzed oxidative cyclization of 2-aminobenzothiazole and 2-phenoxyacetophenones led to 3-phenoxybenzo[*d*]imidazo [2,1-*b*]thiazoles **37a**–**e** in high yield (Scheme 29) [59]. Atmospheric oxygen acted as an oxidant. The ketones with both electron-donor and electron-acceptor substituents readily entered the reaction. Of special interest for synthesizing drugs are *C*-3 oxo-substituted imidazoheterocycles.

**Scheme 30:**
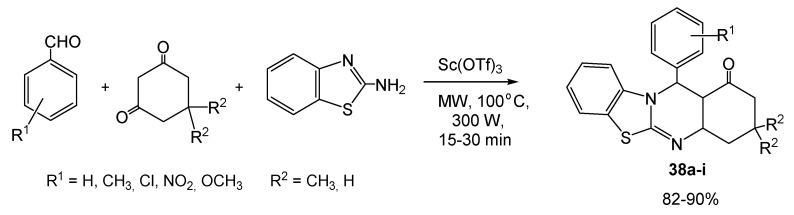
Atom-economy green synthesis of benzothiazole-quinazolinones **38a**–**i**.

An example of green synthesis is Sc(OTf)_3_-catalyzed MW-assisted atom-economy three-component reaction of 2-aminobenzothiazole, aromatic aldehydes and 1,3-diketones, leading to annulated products 38 bearing pharmacophore motifs (Scheme 30) [60]. The reaction proceeds as the CO-activation, Knoevenagel condensation reaction, and nucleophilic addition of azole followed by intramolecular cyclization. The advantages of the reaction, such as high product yields, short time, easy isolation of the products and ecologically safe conditions, can be realized only by a combination of Sc(OTf)_3_ catalysis, MW-assistance, and solvent-free conditions.

**Scheme 31:**
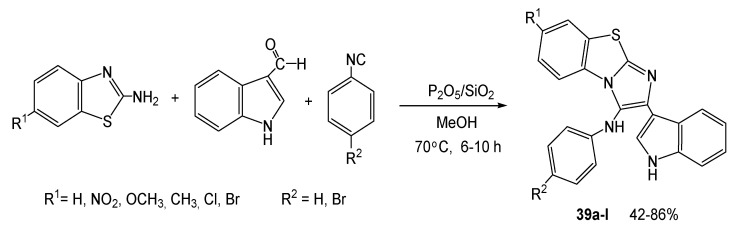
Three-component synthesis of polycyclic 3-aminoimidazo[2,1-*b*](1,3)-benzothiazoles **39a**–**l**.

Another example of an environmentally friendly process is the multicomponent reaction of 2-aminobenzothiazole or its 6-substituted derivatives, indole-3-carbaldehyde and arylisonitriles, in the presence of P_2_O_5_ on SiO_2_ as the acidic catalyst (Scheme 31) [61]. The formation of the products, 3-aminoimidazo[2,1-*b*](1,3)benzothiazoles 39a-l, is facilitated by fast removal of water due to strong dehydrating agent P_2_O_5_/SiO_2_. This catalyst is widely used nowadays in view of its effectiveness, ecological safety, stability, low toxicity and simple handling.

**Scheme 32:**
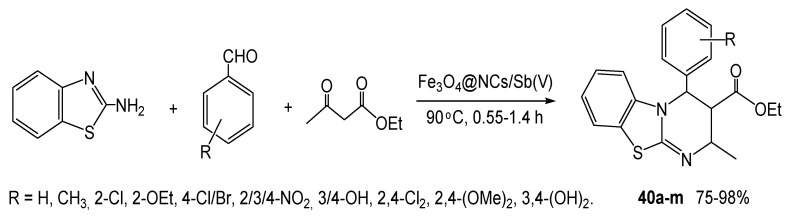
Three-component synthesis of 4*H*-pyrimido[2,1-*b*]benzothiazoles **40a**–**m**.

Recently, the use of the cellulose-based nanocatalyst Fe_3_O_4_@NCs/Sb(V) to synthesize 4*H*-pyrimido[2,1-*b*]benzothiazoles **40a**–**m** was described [62]. The one-pot reaction proceeds without solvent at heating (Scheme 32). The yield and the rate of the process are strongly affected by the nature and position of the substituent, the reactivity and the yield being higher for aldehydes with electron-withdrawing groups. The role of the Lewis catalyst is to activate the carbonyl groups and, thus, accelerate the reaction. Up to five turnovers of the catalyst are possible without notable loss of the catalytic activity.

**Scheme 33:**
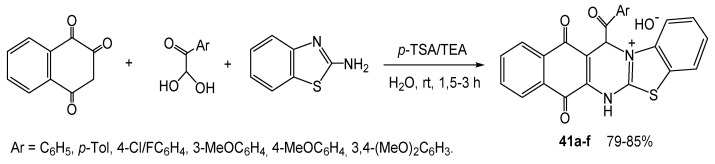
Three-component synthesis of quinazoline derivatives of 2-aminobenzothiazole **41a**–**f**.

A similar approach, but with *p*-TSA as a catalyst, was used to synthesize quinazoline derivatives of 2-aminobenzothiazole **41a**–**f** [63]. The three-component reaction proceeds under mild conditions (aqueous acetone, room temperature) and includes a cascade of reactions (Scheme 33): Knoevenagel condensation reaction, Michael addition with subsequent intramolecular condensation of the imino group to the carbonyl group of the quinone fragment, and, finally, the intramolecular dehydration/aromatization of the intermediates and the keto-enol rearrangement. *p*-TSA is supposed to accelerate the reaction due to the increase of nucleophilicity of the nitrogen atom in 2-aminobenzothiazole. Mild reaction conditions, readily available catalysts, simple product isolation, good yield (79–85%) provide ecological safety and efficiency of the method.

**Scheme 34:**
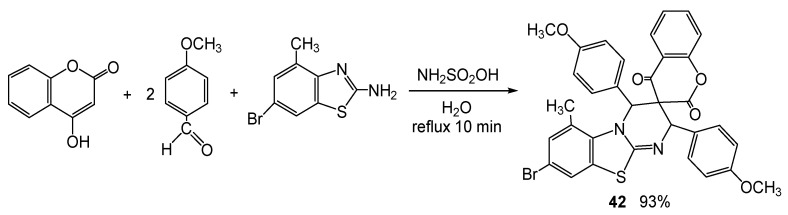
Synthesis of spiroheterocycle **42** from 2-amino-6-bromo-4-methylbenzothiazole, 4-anisaldehydeгидa and 4-hydroxycoumarine.

A strategy for the design of spiroheterocycles annulated with biologically active fragments was developed on the example of the pseudo-four-component reaction of 2-amino-6-bromo-4-methylbenzothiazole, 4-anisaldehyde and 4-hydroxycoumarine in the presence of sulfamic acid [64]. The reaction proceeds in 93% yield in 10 min resulting in the target product **42** (Scheme 34). The proposed mechanism includes the acid-catalyzed sequence of reactions: the Knoevenagel reaction, condensation of the second aldehyde molecule with 2-aminobenzothiazole, the Diels–Alder heterocycloaddition of the intermediates. Spirocyclic structures are privileged in synthetic and medicinal chemistry due to their structural rigidity, which strongly affects biological and pharmacological activity. By the use of different cyclic β-diketones, 2-aminobenzothiazoles and aromatic aldehydes, three series (12 examples) of structurally diverse spiroheterocycles have been synthesized in the aqueous medium in high yields (88–93%).

**Scheme 35:**
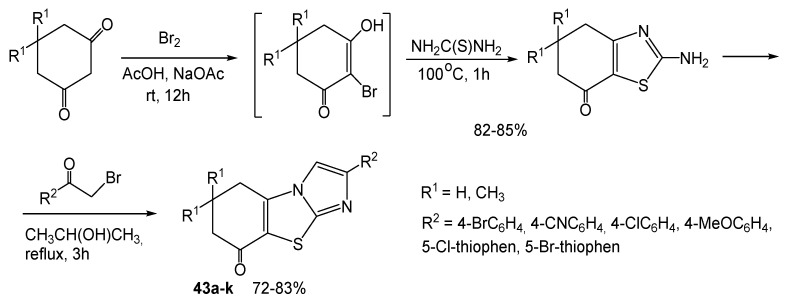
Synthesis of tricyclic derivatives of imidazo[2,1-*b*](1,3)benzothiazolones **43a**–**k**.

Another route to the design of biologically active derivatives of 2-aminobenzothiazole is a successive multistep synthesis based on the commercially available substrates and reagents. Thus, a two-step synthesis of tricyclic derivatives of imidazo[2,1-*b*](1,3)benzothiazolones **43a**–**k** was realized (Scheme 35) [6]. In the first step, 2-aminodihydrobenzothiazole-7-ones were obtained by the reaction of 1,3-diketones with bromine and thiourea in situ, which were then involved in alkylation with aromatic or heteroaromatic α-halogenoketones with subsequent intramolecular cyclization. The substituent in the imidazole ring is of principal importance for antiviral activity because it was shown that the replacement of the aryl group by a smaller thiophene substituent drastically increases the antiviral action.

**Scheme 36:**
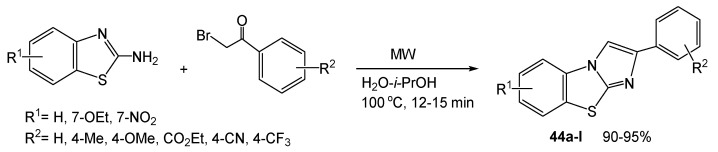
MW-assisted, catalyst-free synthesis of benzo[*d*]imidazo[2,1-*b*]thiazoles **44a**–**l**.

Catalyst-free MW-assisted method for preparation of annulated heterocyclic structures in aqueous medium was demonstrated by the synthesis of benzo[*d*]imidazo[2,1-*b*]thiazoles **44a**–**l** (Scheme 36) [65]. As a polar protic “green” co-solvent capable of easy absorbing MW-radiation, isopropanol was used. The yields were excellent (90–95%) irrespective of the substituents in the benzene ring. The advantages of MW irradiation combined with the use of aqueous isopropanol include short time, the absence of side products, excellent yields, no toxic catalysts, and wide spectrum of the substrates, scalability, thus proving that the process is economic and environmentally friendly. The reaction proceeds successively via the in situ steps and includes quaternization of 2-aminobenzothiazole with bromomethyl aryl ketone, HBr evolution, and formation of the imine with subsequent intramolecular cyclization. The final step is water elimination and aromatization. As a rule, condensed heterocyclic systems possess various types of biological activity.

### 2.4. 2-Hydrazinobenzothiazoles as Analogs of 2-Aminobenzothiazoles

**Scheme 37:**
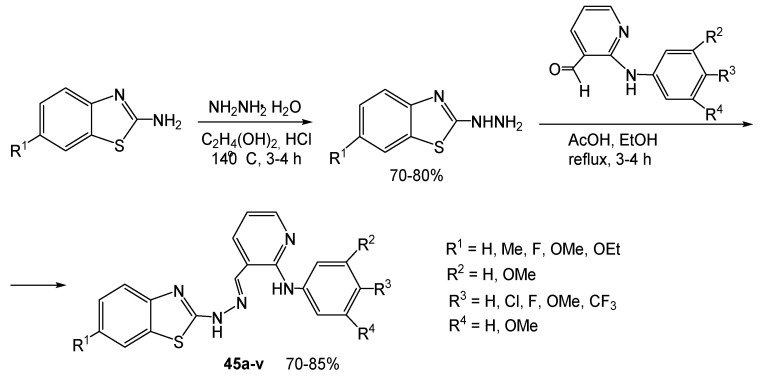
Synthesis of benzothiazole hydrazones with 2-anilinopyridyl groups **45a**–**v**.

Replacement of the amino group in 2-aminobenzothiazoles by the hydrazine residue opens the way to numerous hydrazones whose chemistry is thoroughly studied. An example of such an approach was demonstrated by the synthesis of 6-substituted 2-hydrazinobenzothiazoles by the reaction of the corresponding 2-aminobenzothiazoles with hydrazine hydrate followed by the reaction with various 2-(arylamino)nicotinealdehydes (Scheme 37) [34]. The products **45a**–**v** possess antiproliferative activity. Both the benzothiazole and aniline pharmacophores can have both electron-donor and electron-acceptor substituents. The highest antitumor effect was observed for compounds having electron-donor 6-substituents in the benzothiazole fragment and electron-acceptor substituents in the aniline phenyl ring.

**Scheme 38:**
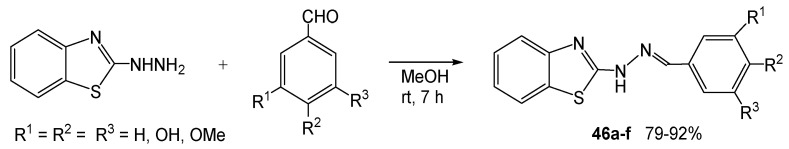
Synthesis of antimalarial 2-benzothiazolyl hydrazones **46a**–**f**.

Another example is the synthesis of antimalarial drugs **46a**–**f** from 2-hydrazinobenzothiazole (Scheme 38) [37]. The structure–activity relationship (SAR) investigation showed that it is the N,S-containing five-membered aromatic ring in the benzothiazole hydrazones, which may be responsible for the antimalarial activity.

**Scheme 39:**
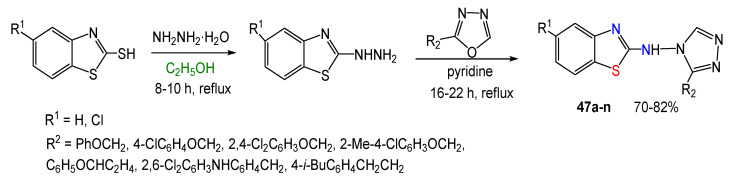
Synthesis of benzothiazole-triazole compounds **47a**–**n**.

To finalize this part and to transfer the bridge to the next section, let us consider several works in which the derivatives of 2-hydrazinobenzothiazole were obtained from 2-mercaptobenzothiazole, and the hydrazine residue was further functionalized. Thus, in [22,23], the target products were obtained by the reflux of mercapto benzothiazoles with hydrazine hydrate in ethanol (Scheme 39). Condensation of the formed 2-hydrazinobenzothiazoles with oxadiazoles in dry pyridine led to 1,2,4-triazoles **47a**–**n** in good yield via the ring-opening/ring-closure reaction.

**Scheme 40:**
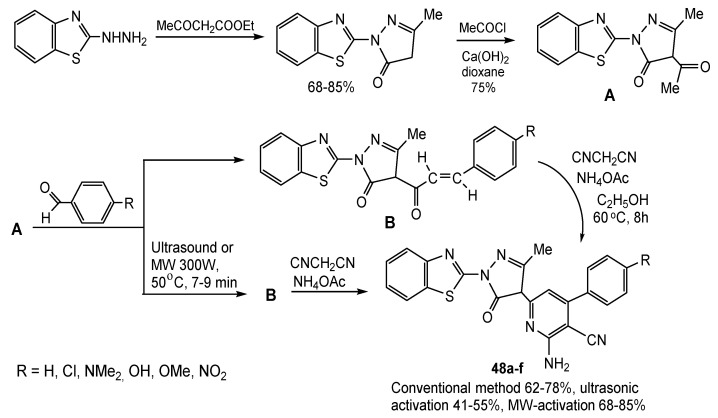
Synthesis of 2-aminonicotine nitriles **48a**–**f** having the benzothiazole fragment.

Conversion of the hydrazine moiety in 2-hydrazinobenzothiazole to the pyrazole ring and further functionalization of the latter was realized, as shown in Scheme 40 [66]. 2-Hydrazinobenzothiazole, prepared as in Scheme 39, reacted with ethyl acetoacetate to give benzothiazolo-5-pyrazolone (A). The latter was converted to the derivatives of 2-aminonicotine nitrile bearing the benzothiazole motif by a different two-step procedure: by a conventional method, ultrasound, and MW activation. The ultrasound activation was less effective (68% yield), whereas, for conventional or MW-assisted syntheses, the yields in the first step reached 83 and 85% of intermediate B, respectively. Further reaction of the intermediate B with malononitrile in the presence of ammonium acetate to give the 2-aminonicotine nitriles **48a**–**f** in good yields. The decisive advantage of the MW irradiation was the short reaction time and higher yields.

Therefore, 2-aminobenzothiazole and its derivatives are of great synthetic potential for synthesizing various heterocyclic systems, including fused and spirocyclic compounds. Recent research in this field achieved substantial progress in discovering new benzothiazolium compounds as good candidates for drugs with different biological activity. This activity may strongly depend on the nature and position of the substituents both in the benzene ring of the benzothiazole moiety and in the heterocycles formed by the functionalization of the amino group.

## 3. 2-Mercaptobenzothiazoles

### Synthesis

As 2-aminobenzothiazoles, 2-mercaptobenzothiazoles attract the interest of researchers due to their various biological activities viz. antimicrobial [18,19,66,67,68,69], anti-inflammatory and antioxidant [69,70,71], antitumor [72,73,74]. Agrochemicals with 2-mercaptobenzothiazole moiety in the molecule are used as fungicides of a wide spectrum [75,76,77], herbicides [78], insecticides [39,78,79]. In industry, they are used as metal corrosion inhibitors in different media [80,81,82,83,84,85,86,87,88,89,90], additives to lubricants [89,90], sorbents of trace amounts of metals, including noble metals [91,92,93] and rubber vulcanization accelerators [94,95,96].

Not only organic derivatives of 2-mercaptobenzothiazole, but also its organosilicon analogs are valuable reagents and synthetic building blocks; they occupy an important place in the chemistry of polymers and materials [88,93,97]. The introduction of organosilicon groups containing biogenic elements in the molecule of 2-mercaptobenzothiazole can render new properties to the compounds. However, organosilicon thiol derivatives of azoles are still poorly studied.

**Scheme 41:**
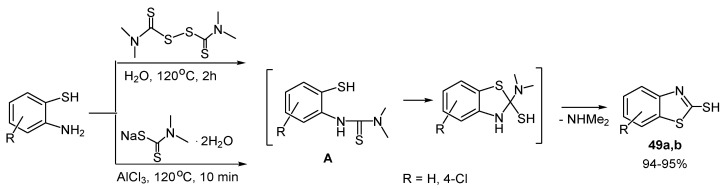
2-Mercaptobenzothiazoles **49a**,**b** from aminothiophenols and tetramethylthiuram disulfide or sodium dimethyl dithiocarbamate.

A simple, efficient, catalyst-free method for synthesizing precursors of potentially biologically active derivatives of 2-mercaptobenzothiazole **49a**,**b** in excellent yield was developed via cyclization of 2-aminothiophenols with tetramethyl thiuram disulfide in water by heating on an oil bath (Scheme 41) [98]. In an alternative modified procedure [99], sodium dithiocarbamate in DMF was used as a reagent and AlCl_3_ as the catalyst. Apparently, the reaction proceeds via the intermediate formation of thiourea A, which suffers intramolecular cyclization with the elimination of dimethylamine and the formation of the target products (Scheme 41).

**Scheme 42:**
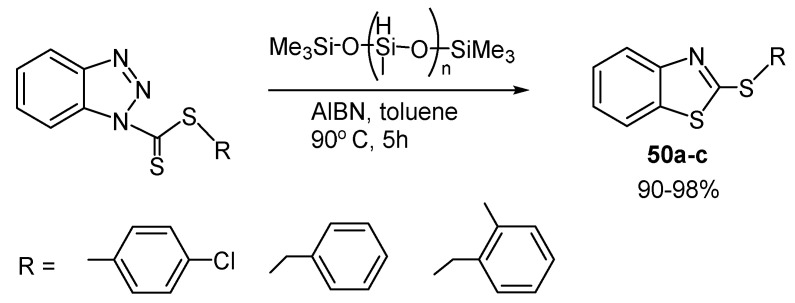
Mercaptobenzothiazoles **50a**–**c** from *N*-thioacylbenzotriazoles in the presence of PMHS.

Green chemistry synthesis of 2-arylthiobenzothiazoles 50a–c was described in [100]. The ring-opening of the five-membered cycle of benzotriazole in aryl 1*H*-1,2,3-benzotriazole-1-carbodithioates and the subsequent cyclization was performed by the use of polymethylhydrosiloxane (PMHS) acting both as the solvent and the reagent in the presence of AIBN as an initiator (Scheme 42). Earlier, in this reaction, a highly toxic tributyltin hydride *n*-Bu_3_SnH was used. The use of PMHS, which is a byproduct in the synthesis of silicone, makes this approach green and practical, which can be considered as an industrial method to synthesize benzothiazoles.

**Scheme 43:**
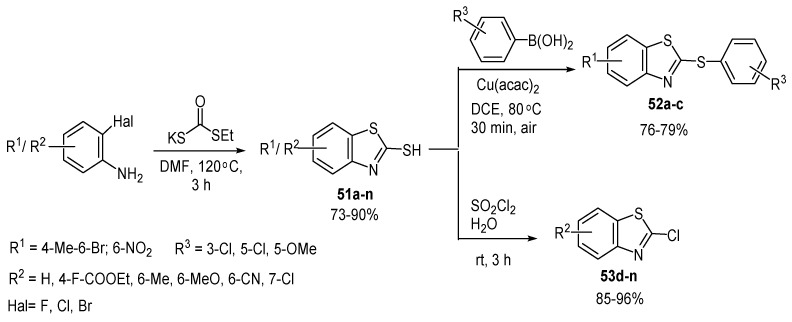
Synthesis of precursors of 2-mercaptobenzothiazoles **51a**–**n** from 2-halogenoanilines and potassium xanthogenate and their further functionalization.

Different 2-mercaptobenzothiazoles **51a**–**n** can be readily prepared from the easily available 2-halogenoanilines and potassium xanthogenate (Scheme 43). Compounds **51a**–**c** prepared in [101] were subjected to cross-coupling with aryl boronic acids in dichloroethane (DCE) in the presence of Cu(acac)_2_ (Scheme 43, upper reaction). The method is characterized by mild conditions, the absence of additional ligand, short reaction time and good yield. The reaction does not depend on the substituents in the reagents.

Later on [102], this method was applied to the synthesis of 2-mercaptobenzothiazoles **51d**–**n** (Scheme 43). Their treatment with sulfuryl chloride gave 2-chlorobenzothiazoles **53d**–**n** in excellent yield. Earlier, this simple procedure gave low yields and was poorly reproducible. In [102], the authors have found that the simple addition of water substantially increases the effectiveness of the reaction. This effect was assigned to the formation of acid by partial hydrolysis of sulfuryl chloride. The formed 2-chlorobenzothiazoles **53d**–**n** act as precursors in the synthesis of 2-substituted benzothiazoles playing an important role in medicinal chemistry due to their pharmacological properties.

**Scheme 44:**
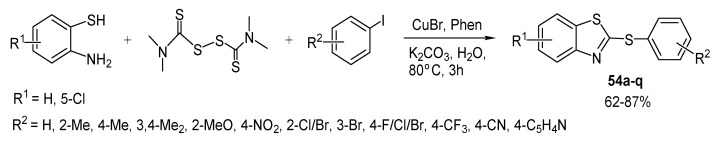
CuBr-catalyzed synthesis of 2-mercaptobenzothiazoles **54a**–**g** from *o*-aminothiophenols, tetramethylthiuram disulfide and iodobenzenes.

The tandem reaction for synthesizing aryl derivatives of 2-mercaptobenzothiazole **54a**–**g** was reported (Scheme 44) [103]. The in situ inter- and intramolecular condensation of *o*-aminothiophenols with tetramethylthiuram disulfide gives rise to the corresponding 2-mercaptobenzothiazoles, which further underwent intermolecular coupling with iodobenzenes. Among the studied copper catalysts, CuBr used at 80 °C was found to be the most effective. Other metal catalysts, such as Fe, Co, or Ni, were ineffective as catalysts. The derivatives of 2-mercaptobenzothiazole with F, Cl, Br as substituents in the aryl ring were obtained in high yield (76–84%). Therefore, the use of inexpensive catalysts, simple ligand, water as the solvent, and moderate reaction temperature make the process practically valuable and useful in organic synthesis.

**Scheme 45:**
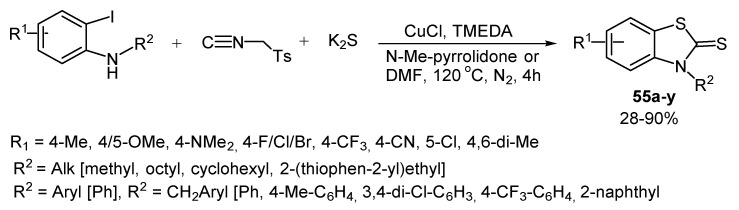
Benzothiazolethiones **55a**–**y** from *o*-iodoanilines, K_2_S and (tosylmethyl)isocyanide.

The CuCl-catalyzed three-component reaction of *o*-iodoanilines, K_2_S and (tosylmethyl)isocyanide proceeds with the formation of two C–S and one C=S bond and gives rise to benzothiazolethiones **55a**–**y** (Scheme 45) [104]. The reaction is influenced by the substituents in both the ring and at the nitrogen.

**Scheme 46:**
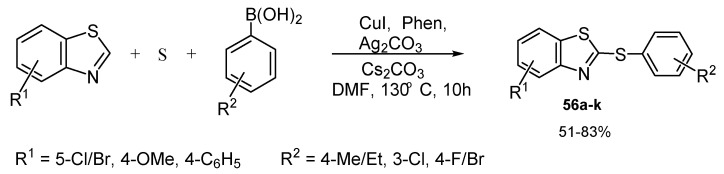
Oxidative synthesis of 2-arylthiobenzothiazoles from benzothiazoles, elemental sulfur and arylboronic acids.

The result of a one-pot, CuI-catalyzed reaction of benzothiazoles, sulfur and aryl boronic acids were shown to strongly depend on the nature of the oxidant (Scheme 46) [105]. No reaction or only trace amounts of the target products **56a**–**k** were obtained with a number of oxidants examined. Among different silver salts, Ag_2_CO_3_ demonstrated the highest oxidative activity.

The above methods of assembling the 2-mercaptobenzothiazoles via direct functionalization of the C2–H bond, although being atom-economic and ecologically pure, suffer from such drawbacks as the reaction temperatures up to 120–140 °C, the use of stoichiometric amounts of copper catalyst, a strong oxidant, and, in some cases, specific ligands.

**Scheme 47:**
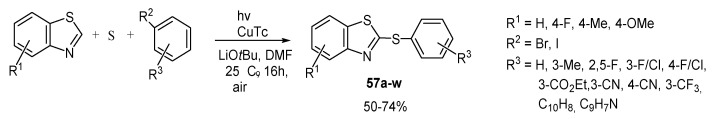
Synthesis of 2-(arylthio)benzothiazoles **57a**–**w** via photoinduced sulfanylation.

The alternative approach is the photoinduced sulfanylation of the C2–H bond in benzothiazoles by aryl(hetaryl) electrophiles and elemental sulfur. The reaction shown in Scheme 47 occurs at room temperature in air in the presence of copper(I) thiophene-2-carboxylate (CuTc) [106] and can be applied to the synthesis of a wide series of alkyl(aryl) or hetaryl derivatives **57a**–**w**. The photocatalysis proceeds via the in situ formation of diaryl disulfides as key intermediates.

**Scheme 48:**
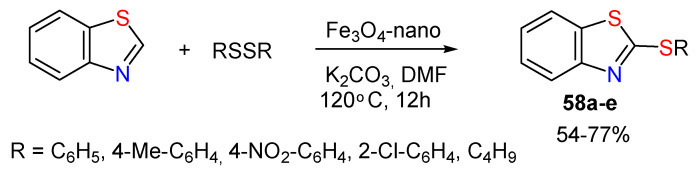
Nano-Fe_3_O_4_ promoted synthesis of 2-(arylthio)benzothiazoles **58a**–**e**.

Direct sulfanylation of the C2–H bond in benzothiazole by disulfides using nanosized Fe_3_O_4_ particles was realized to prepare 2-(arylthio)benzothiazoles **58a**–**e** (Scheme 48) [107]. Apparently, nanosized powdered catalyst Fe_3_O_4_ acts as the Lewis acid by activating the disulfide via the S–S bond splitting. The formed anion ([Fe_3_O_4_]SPh)^–^ is oxidized by air oxygen to recover the initial disulfide and the nanosized catalyst so that half of the molar equivalent of disulfide is required. The advantages of the method are the atom-economy synthesis, non-inert atmosphere, small quantities of the highly efficient catalyst, and recycling.

**Scheme 49:**
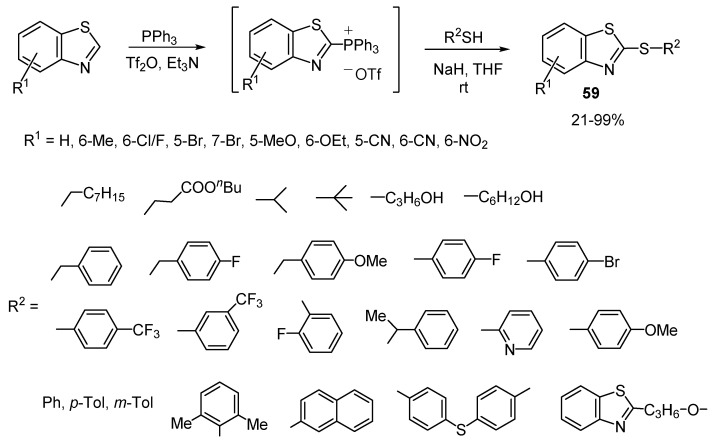
The synthesis of benzothiazol-2-yl sulfides **59** from benzothiazole, triphenylphosphine and S-nucleophiles.

An interesting example of C2–H-functionalization of thiazoles using phosphines was reported recently [108]. The intermediate triflate phosphonium salts (Scheme 49) were treated with thiols, which form the corresponding thiolates by treatment with sodium hydride. The reactions proceed under mild conditions; the starting triphenylphosphine can be recovered, the yield varied from low (21%) to quantitative (99%). Remarkably, electronic or steric factors do not have a notable effect on the efficiency of the reactions.

**Scheme 50:**
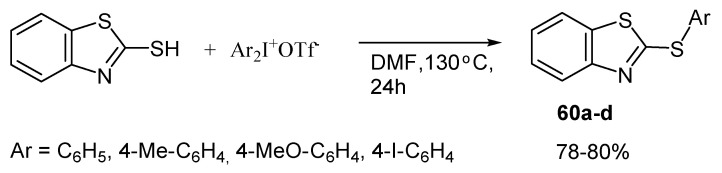
Base-free S-arylation of 2-mercaptobenzothiazole with diaryliodonium triflates.

Functionalization of the SH group is the key step in the synthesis of 2-mercaptobenzothiazole derivatives with pronounced biological activity. Thus, aerobic base-free and transition metal catalyst-free regioselective S-arylation of 2-mercaptobenzothiazole with diaryliodonium triflates in DMF at 130 °C with the formation of 2-(arylthio)benzothiazoles **60a**–**d** in good yields was reported (Scheme 50) [109].

**Scheme 51:**
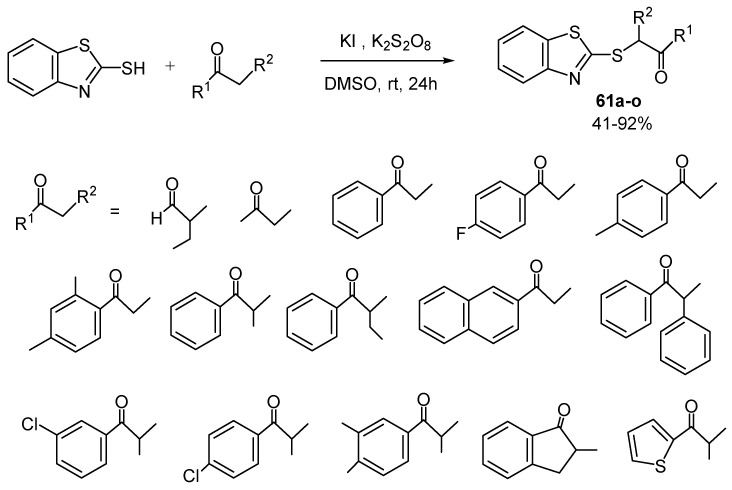
KI/K_2_S_2_O_8_-promoted S-acylmethylation of 2-mercaptobenzothiazole with ketones.

The KI/K_2_S_2_O_8_-mediated C–H sulfanylation of ketones with 2-mercaptobenzothiazole easily occurs at room temperature, leading to various β-ketothio esters **61a**–**o** (Scheme 51) [110]. The yields for aromatic ketones were 67–92%, with aliphatic ketone or aldehyde, they decreased to 41 and 47%.

**Scheme 52:**
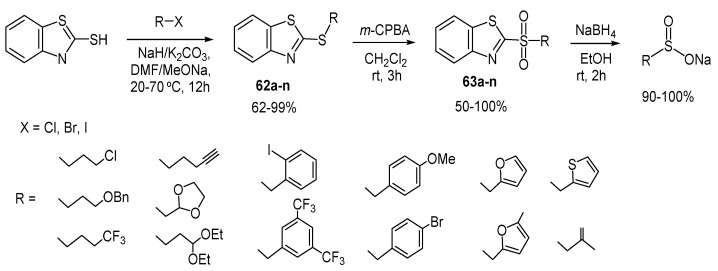
Sequence of reactions 2-mercaptobenzothiazole → thioethers **62a**–**n** → sulfones **63a**–**n** → sulfinate salts.

The two-step protocol was proposed for synthesizing 2-benzothiazolyl sulfones **63a**–**n** via intermediate thioethers **62a**–**n** and their oxidation with *m*-CPBA (Scheme 52) [111]. Sulfones **63a**–**n** were reduced with sodium borohydride to afford functionalized sulfonates—universal intermediates in organic, biopharmaceutical and polymer chemistry—in up to quantitative yield.

**Scheme 53:**
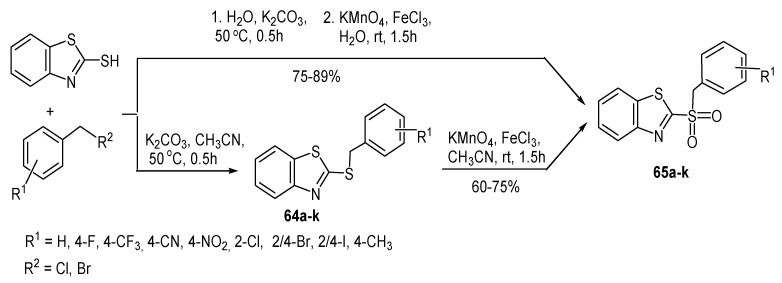
Different protocols for synthesizing biologically active sulfones **65a**–**k**.

Alkylation of 2-mercaptobenzothiazole with benzyl halogenides and oxidation of the formed sulfides **64a**–**k** by KMnO_4_ in the presence of FeCl_3_ catalyst to sulfones **65a**–**k** was realized in two different ways: in two separate steps with isolation of intermediate sulfides **64a**–**k** or in one-pot reaction (Scheme 53) [76,77]. The use of water as the solvent, inexpensive oxidant and higher yields in one-pot reaction make the process to green protocols. The antifungal activity of sulfones **65a**–**k** is much higher than that of their non-oxidized precursors **64a**–**k**, probably, due to the higher hydrophilicity of the former, increasing their ability to penetrate through biological membranes.

Using the same procedure as in the first step of the reaction of benzothiazole with a series of substituted benzyl chlorides in the presence of KI, a series of aryl sulfides of general formula **64** was synthesized and shown to have antiparasitic, anti-inflammatory and antioxidant activity [79].

**Scheme 54:**
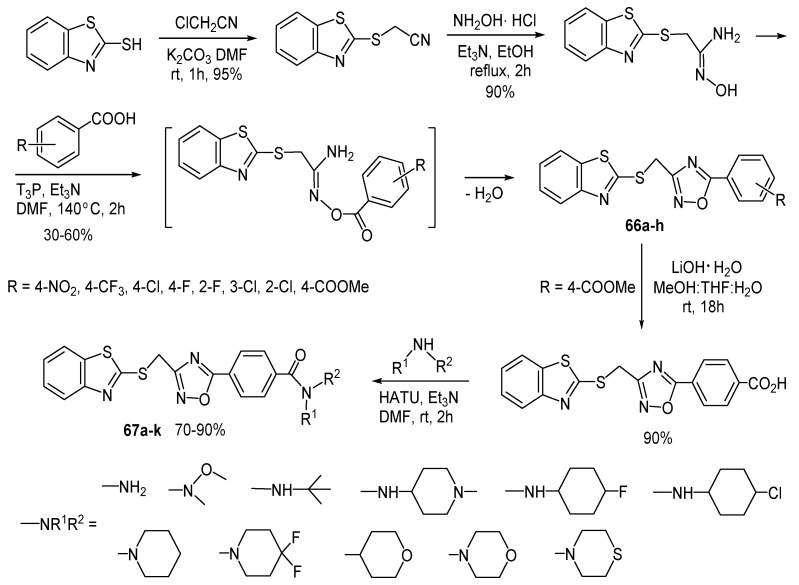
Multistep synthesis of anti-inflammatory mercaptobenzothiazole-1,2,4-oxadiazoles **66a**–**h** and their benzamide analogs **67a**–**k**.

A similar reaction of cyanomethylation of the SH group followed by hydroxyamination of the formed nitrile and further functionalization led to a series of mercaptothiazole-1,2,4-oxadiazole compounds **66a**–**h**, which demonstrated better anti-inflammatory activity than ibuprofen (Scheme 54) [70]. Note that the method is equally successful for benzoic acids containing electron-withdrawing or electron releasing groups in the ring. Methyl benzoate (R = 4-COOMe) formed in the maximal yield was used to synthesize the amide analogs **67a**–**k** (Scheme 54, T_3_P = propylphosphonic anhydride; HATU = hexafluorophosphate azabenzotriazole tetramethyl uronium) [70].

**Scheme 55:**
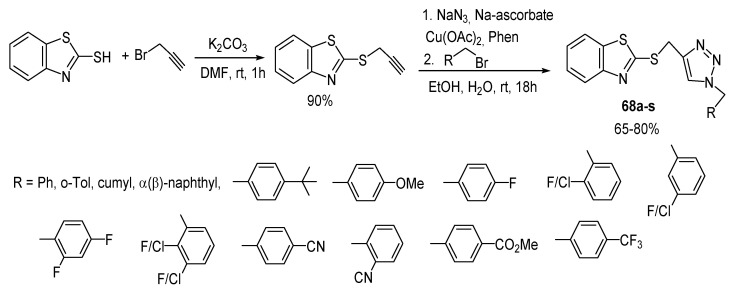
Synthesis of benzothiazole-1,2,3-triazole compounds **68a**–**s**.

From 2-mercaptobenzothiazole and propargyl bromide, 2-propargyl thiobenzothiazole was obtained, which reacted with sodium azide and benzyl bromides to give benzothiazole-1,2,3-triazoles **68a–s** (Scheme 55 [71]. Benzyl bromides with both electron-donor and electron-acceptor substituents react in good yield.

Compound **68** with R = C_6_H_4_COOMe was hydrolyzed to the corresponding acid **69** (R = C_6_H_4_COOH) and amide 70 (R = C_6_H_4_CONH_2_), which both showed antitumor effect [71].

**Scheme 56:**
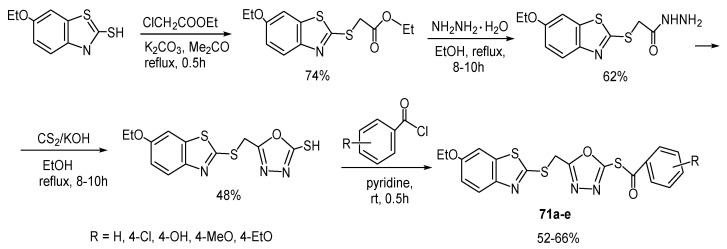
Multistep synthesis of benzothiazole-1,3,4-oxadiazole heterocycles **71a**–**e** having anti-inflammatory, antimicrobial and antioxidant activity.

A series of compounds having anti-inflammatory, antimicrobial and antioxidant activity and possessing the benzothiazole and 1,3,4-oxadiazole heterocycles **71a**–**e** was synthesized starting from 6-ethoxy-2-mercaptobenzothiazole by alkylation with ethyl chloroacetate, hydrazinolysis, cyclization with carbon disulfide and, finally, S-acylation (Scheme 56) [69].

**Scheme 57:**
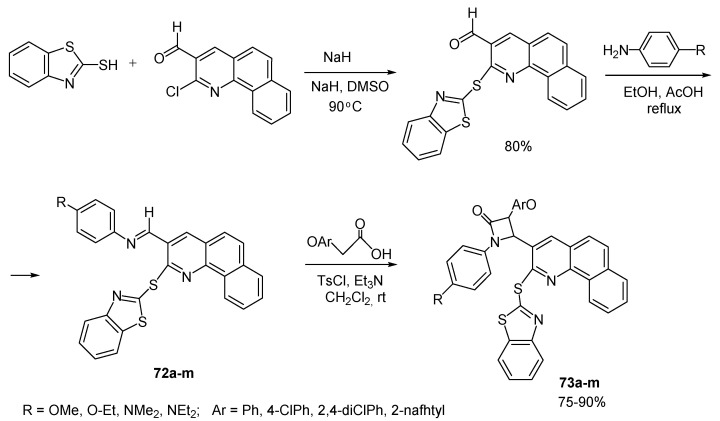
Synthesis of lactames **73a**–**m** and their precursors **72a**–**m** from 2-mercaptobenzothiazole.

The design, synthesis and biological activity study of β-lactames possessing benzoquiniline and 2-mercaptobenzothiazole heterocycles **73a**–**m** was performed. First, the reaction of arylation gave the corresponding aldehyde an 80% yield, which reacted with anilines to give imines **72a**–**m**. The [2+2]-cycloaddition reaction of the latter with ketenes generated from substituted acetic acids in the presence of triethylamine and tosyl chloride led exclusively to *cis*-β-lactames **73a**–**m** in high yield (Scheme 57) [67]. β-Lactames **73a**–**m** and their imine predecessors **72a**–**m** showed high antibacterial activity with low toxicity.

**Scheme 58:**
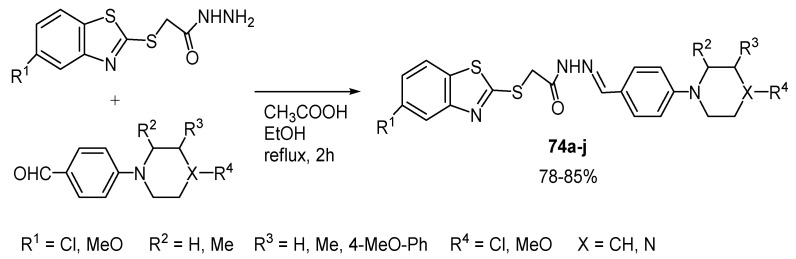
Synthesis of benzothiazolyl acyl hydrazones **74a**–**j** with antitumor activity.

Acylhydrazides based on 5-substituted 2-mercaptobenzothiazoles react with *p*-hetaryl-substituted benzaldehydes to afford benzothiazolyl acyl hydrazones **74a**–**j** having an antitumor activity (Scheme 58) [74].

**Scheme 59:**
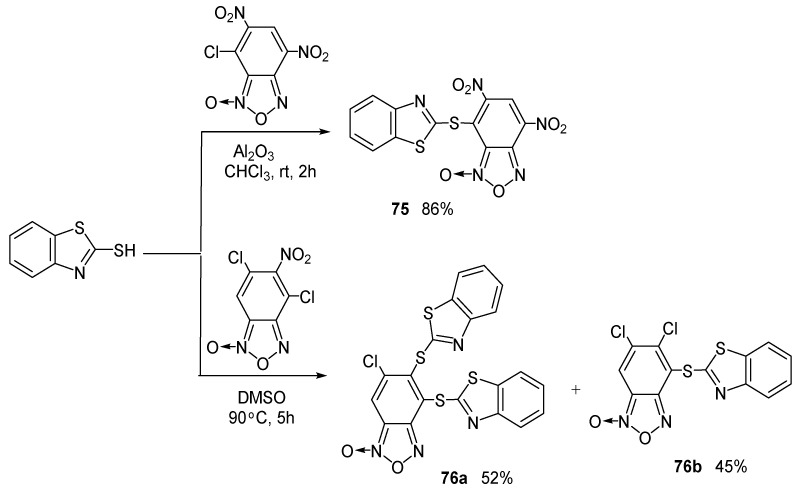
Synthesis of scaffolds **75**, **76** from 2-mercaptobenzothiazole and 4,6,7-substituted 2,1,3-benzoxadiazole 1-oxides.

New structural hybrids of benzofuroxan and benzothiazole derivatives were synthesized by nucleophilic aromatic substitution in 7-chloro-4,6-dinitro-2,1,3-benzoxadiazole 1-oxide or 4,6-dichloro-5-nitro-2,1,3-benzoxadiazole 1-oxide by 2-mercaptobenzothiazole [19]. Remarkably, with the former, the reaction proceeds as substitution of the chlorine atom activated by three nitro groups (two in the benzene ring and one in the 1,2,5-oxadiazole 2-oxide moiety) to give compound **75**, while with the latter, either two thiol residues appear in the 4- and 5-positions (compound **76a**), or chlorine atom replaces the 5-NO_2_ group, and the thiol residue goes to the 4-position (compound **76b**, Scheme 59). The synthesized scaffolds **75** and **76** are considered pro-drugs that realize their biological activity via the intracell mediators [19].

**Scheme 60:**
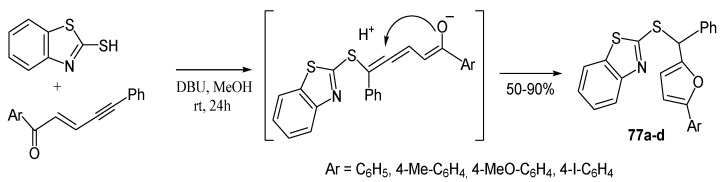
Benzothiazolyl furfuryl sulfides **77a**–**d** via cyclization of 2-mercaptobenzothiazole with aryl enyne ketones.

2-Mercaptobenzothiazole reacts with aryl enyne ketones in the presence of DBU at room temperature via cyclization to give benzothiazolyl furfuryl sulfides **77a**–**d** [112]. Cyclization to furans proceeds, apparently, via the intermediate ions (Scheme 60). Ketones with electron-acceptor substituents in the aryl ring give higher yields of the products. Compounds **77** are potential fungicides.

**Scheme 61:**
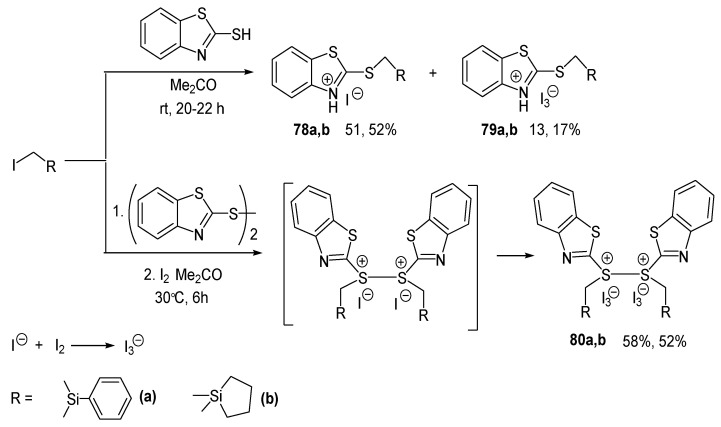
Organosilicon salts **78**, **79** and disulfonium dications **80** from 2-mercaptobenzothiazole.

In a series of our works, the reactions of 2-mercaptobenzothiazole with iodomethylsilanes were examined. As distinct from the reactions described above, 2-mercaptobenzothiazole, when treated with (iodomethyl)(dimethyl)phenyl silane (a) or (iodomethyl)-1-methylsilolane (b), having the exocyclic or endocyclic silicon atom in the molecule, afford new iminium salts **78a**,**b** and **79a**,**b** with the iodide or triiodide counter-ions (Scheme 61) [113]. The reaction proceeds at room temperature in the absence of bases or phase-transfer catalysts. When carrying out the reaction in the presence of equimolar amounts of iodine required for the formation of triiodide anion, the yield of compounds **79a**,**b** is increased by three times. New ionic liquids having stable organosilicon disulfonium cations and iodide and triiodide counter-ions **80a**,**b** were obtained by the reaction with di(2-benzothiazolyl)disulfide. Compounds **80** are promising electrophiles for synthesizing organoelement derivatives by the reaction with C-nucleophiles. The increased interest in the salts of heterocyclic compounds is due to wide application in different fields [114,115,116,117,118,119].

**Scheme 62:**
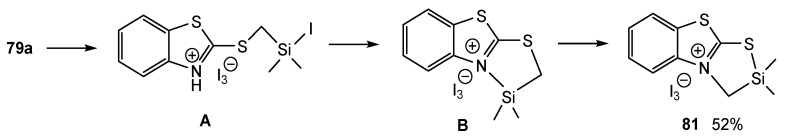
Annulation with rearrangement of salt **79a** to salt **81**.

The three-component reaction of 2-mercaptobenzothiazole, silane (a, see Scheme 61) and molecular iodine, in addition to product **79a**, leads to the annulated N,S,Si-heterocycle **81** [120]. The formation of this interesting tricyclic structure occurs due to partial splitting of the Si-C_sp2_ in the product of S-alkylation **79a** induced by the in situ formed HI and elemental iodine. The intermediate labile silane A readily enters the reaction of intramolecular cycloquaternization resulting in the formation of B, which suffers the intramolecular rearrangement with the migration of the methylene group in the N–Si–CH_2_–S в N–CH_2_–Si–S in the heterocycle to give salt 81 (Scheme 62). To the best of our knowledge, this is the first example of the annulation of 2-mercaptothiazolyl derivative resulting in the so-far unknown 2,3-dihydro[1,4,2]thiazasilolo[5,4-*b*][1,3]benzothiazol-4-ium heterocyclic system.

**Scheme 63:**
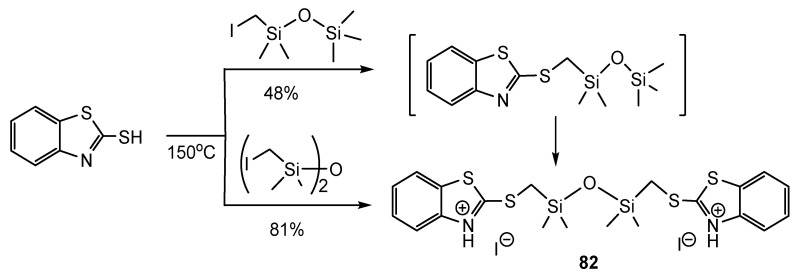
Synthesis of bis-iminium salt **82** from 2-mercaptobenzothiazole and iodomethylsiloxanes.

2-Mercaptobenzothiazole was S-alkylated with mono- and bifunctional iodomethylsiloxanes at high temperatures in the solvent-free and base-free manner to give bis-iminium salt **82** (Scheme 63) [121]. The formation of product **82** from 1-(iodomethyl)-1,1,3,3,3-pentamethyldisiloxane occurs via the splitting of the siloxane bonds in the product of S-alkylation induced by the in situ formed HI. Subsequent intermolecular condensation of the intermediate labile products affords diiodide **82**.

**Scheme 64:**
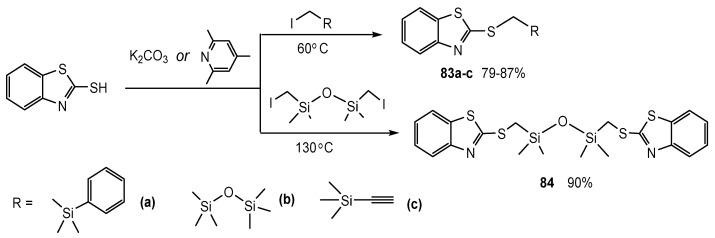
Synthesis of non-salt forms of silicon-aromatic, silicon-acetylenic, and siloxane derivatives of 2-mercaptobenzothiazole **83a**–**c**, **84**.

The reactions of 2-mercaptobenzothiazole with different organosilicon alkylated reagents were also carried out under the conventional conditions, excluding the formation of hydrogen iodide in the reaction mixture and, hence, the bond splitting [121,122]. The reaction is performed in the presence of an inorganic (K_2_CO_3_) or organic non-nucleophilic base (2,4,6-collidine). In this case, non-salt forms of silicon-aromatic, silicon-acetylenic, or siloxane derivatives of 2-mercaptobenzothiazole **83a**–**c**, **84** were obtained (Scheme 64). From the virtual screening using the PASS program, compounds **83a**,**c** in high probability may possess biological activity, displaying antisclerotic and antioxidant activity.

Noteworthy is the presence of tetramethylsiloxane groups in molecules **82**, **83a**, **84** imparting the materials on their basis elasticity, strength, chemical inertness and biocompatibility.

**Scheme 65:**
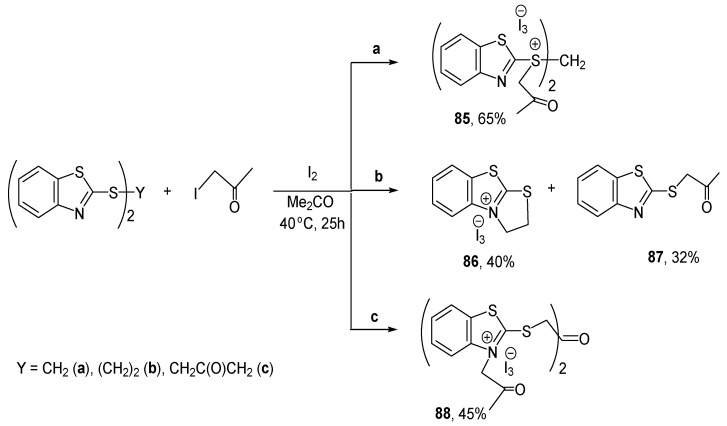
Acylalkylation and annulation reactions of 2-mercaptobenzothiazole derivatives with iodoacetone.

New compounds **85**–**88** (Scheme 65) were obtained by the reaction of 2,2′-(organyldithio)dibenzothiazoles with iodoacetone in the presence of elemental iodine [123]. The presence of I_2_ is necessary for the formation of triiodide counter-ions, which were shown by us to stabilize the formation and accumulation of disulfonium cations.

The course of the reaction is affected by the nature of the spacer introduced in the disulfide link. In the case of polymethylene spacer, the reaction proceeds exclusively on the exocyclic sulfur atoms, whereas the carbonyl group in the spacer initiates the reaction on the nitrogen atoms in the heterocycle.

**Scheme 66:**
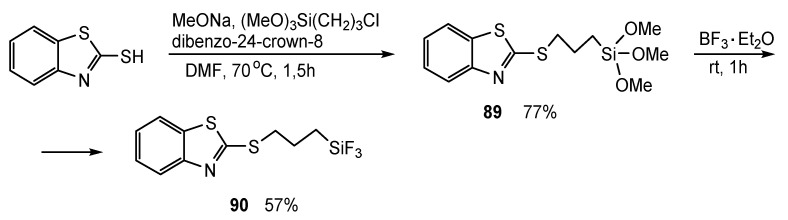
Synthesis of trimethoxysilyl and trifluorosilyl derivatives of 2-mercaptobenzothiazole.

Silylpropyl derivatives of 2-mercaptobenzothiazole have also been described [124]. They have been obtained by nucleophilic substitution of chlorine in (chloropropyl)trimethoxysilane in the presence of sodium methoxide and crown-ether in DMF on heating and gave product 89 in 77% yield. The latter was converted to hydrolytically unstable trifluorosilyl derivative **90** by the reaction of **89** with trifluoroboron etherate (Scheme 66).

**Scheme 67:**
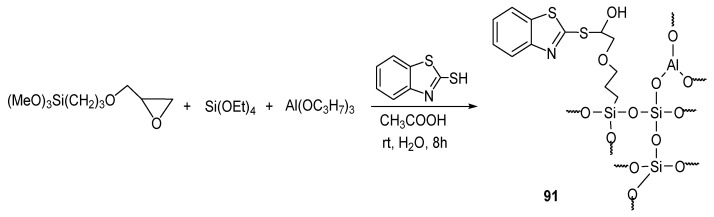
Sol–gel synthesis of anticorrosion coating **91** based on 2-mercaptobenzothiazole.

The sol–gel technique, which is a promising method in material science, was used for the preparation of a silane-based coating **91** using (3-glycidoxypropyl) trimethoxysilane, tetraethyl orthosilicate, and Al(OPr-*i*)_3_ as a chemical modifier, and benzotriazole or 2-mercaptobenzothiazoleas as corrosion inhibitors (Scheme 67) [88].

**Scheme 68:**
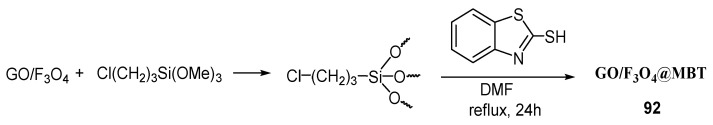
Preparation of nanosorbent **92** based on magnetic GO and 2-mercaptobenzothiazole.

Also, note the use of 2-mercaptobenzothiazole for the preparation of nanocomposite **92** from magnetic graphene oxide (GO/Fe_3_O_4_) and (3-chloropropyl)trimethoxysilane (Scheme 68) [94]. Nanosorbent **92** is successfully used in different matrices for the extraction of trace amounts of cadmium copper and lead.

Consequently, 2-mercaptobenzothiazole and its derivatives, as 2-aminobenzothiazole, have great potential in synthetic organic chemistry, the chemistry of drugs and material chemistry. The results of the last five years have unequivocally proven that investigation in this field allows discovering new reactions and approaches to valuable products of considerable interest as biologically active compounds, new synthons and materials. Functionalization of the amino or mercapto groups in the 2-position allows not only preparing new products but also assembling new types of annulated heterocycles.

## 4. Conclusions

In summary, based on the analysis of the recent literature (since 2015) on the synthesis and chemical transformations of the 2-amino and 2-mercapto-substituted derivatives of benzothiazole in the present review, we conclude that practically all products clearly demonstrate various practically valuable properties. Easy functionalization of the amino and mercapto groups and the benzene ring in 2-amino- or 2-mercaptobenzothiazole allows considering them as highly reactive synthons for the design and preparation of biologically active and industrially demanded products and materials. Note that, in addition to conventional multistep methods of their syntheses, large efforts are being made to develop environmentally friendly atom economy one-pot methods having high synthetic potential and representing the basis of modern organic synthesis.
